# Long-term oncological outcomes following percutaneous microwave ablation of T1 renal cell carcinoma

**DOI:** 10.1093/bjr/tqaf214

**Published:** 2025-08-21

**Authors:** Luke Glover, Joseph John, Alexander Spiers, Richard Guinness, Thomas Dutton, Andrew Gemmell, Rajinder Virdi, Jonathan Skinner, Moira Anderson, Taona Stainer-Smith, Nicholas Campain

**Affiliations:** Department of Medical Imaging, Royal Devon University Healthcare NHS Foundation Trust, Exeter EX2 5DW, United Kingdom; South West Imaging Training Academy, Plymouth PL6 5WR, United Kingdom; Department of Urology, Royal Devon University Healthcare NHS Foundation Trust, Exeter EX2 5DW, United Kingdom; University of Exeter Medical School, St Luke's Campus, Exeter, EX1 2LU, United Kingdom; Department of Medical Imaging, Royal Devon University Healthcare NHS Foundation Trust, Exeter EX2 5DW, United Kingdom; Department of Medical Imaging, Royal Devon University Healthcare NHS Foundation Trust, Exeter EX2 5DW, United Kingdom; Department of Urology, Royal Devon University Healthcare NHS Foundation Trust, Exeter EX2 5DW, United Kingdom; Department of Medical Imaging, Royal Devon University Healthcare NHS Foundation Trust, Exeter EX2 5DW, United Kingdom; Department of Medical Imaging, Royal Devon University Healthcare NHS Foundation Trust, Exeter EX2 5DW, United Kingdom; Department of Medical Imaging, Royal Devon University Healthcare NHS Foundation Trust, Exeter EX2 5DW, United Kingdom; Department of Urology, Royal Devon University Healthcare NHS Foundation Trust, Exeter EX2 5DW, United Kingdom; Department of Urology, Royal Devon University Healthcare NHS Foundation Trust, Exeter EX2 5DW, United Kingdom; Department of Urology, Royal Devon University Healthcare NHS Foundation Trust, Exeter EX2 5DW, United Kingdom

**Keywords:** kidney cancer, microwave ablation, oncological outcomes, renal cell carcinoma, small renal masses, tumour ablation

## Abstract

**Objectives:**

Incidence of small renal masses (SRMs) including renal cell carcinoma (RCC) is increasing. Standard of care is to offer partial nephrectomy (PN), with tumour ablation (TA) considered an alternative in frail/co-morbid patients. This study aimed to determine whether microwave ablation (MWA) is a safe and effective treatment for selected cases of RCC.

**Methods:**

All MWAs performed at a regional tertiary care centre between October 2016 and April 2024 were prospectively recorded on a database. Data collected included tumour and patient characteristics, complications, and recurrences.

**Results:**

Two hundred and nine MWAs were recorded with median 37 months (interquartile range [IQR] 15.3-59.4 months) follow-up. About 94.7% of patients had ≥12 months of follow-up. The biopsy rate was 92%. Following MWA, 93% of patients had a hospital stay of 1 night. Two Clavien-Dindo grade ≥III complications occurred within 30 days (0.96%). Local and metastatic recurrence rates were 5.9% and 2.7%, respectively.

**Conclusions:**

MWA was a safe, effective treatment for SRMs in this large cohort which included young, fit patients and underwent long-term follow-up. Recovery times were short, with low complication rates and favourable oncological outcomes in biopsy-proven T1 RCC <5 cm.

**Advances in knowledge:**

The current study demonstrates a large, diverse MWA cohort (including T1b tumours) with high biopsy rate, minimal loss to follow-up, and long follow-up period facilitating assessment of long-term oncological outcomes in biopsy-proven RCC. The results support MWA as a safe, effective treatment for cT1a RCC that should be offered to patients as part of shared decision making.

## Introduction

The incidence of renal cell carcinoma (RCC) is rising globally.^1^ Small renal masses (SRMs), including RCC and benign renal lesions, are a heterogeneous group of tumours of <4 cm in diameter which enhance on computerized tomography (CT) imaging.^2^ Standard of care is to offer partial nephrectomy (PN) for cT1 tumours, although there is an increasing role of active surveillance (AS) and tumour ablation (TA) which historically was used as an alternative treatment option in older or co-morbid patients.^3^^,^^4^

Microwave ablation (MWA) is a form of thermal ablation which uses electromagnetic waves to heat and destroy cells by coagulative necrosis. Compared to radiofrequency ablation (RFA), MWA creates higher temperatures in a more concentrated and rapid manner, allowing for generation of a more precise ablative field. However, in contrast to cryoablation (CA), the heat generated during MWA may theoretically render the technique less suitable for central tumours with proximity to the renal collecting system.^5^^,^^6^

Current evidence has found comparable oncological outcomes between MWA and PN in propensity-matched studies,^7^ literature reviews,^8^ and meta-analyses.^9^ Safety of MWA has been demonstrated by the present authors who have previously described the shorter-term complication rates and recurrence data of a large series of MWA for SRMs.^10^ Whereas previously MWA was thought of as an experimental technique, new international guidelines now conclude that MWA has the best evidence of the ablative methods in SRMs.^11^

The present study aims to update on the previously reported oncological and safety outcomes and provide a template of real-world practice to inform future guidelines. Focus was on local recurrence, development of metastatic disease, and complication rates at a specialist regional centre based in the United Kingdom.

## Patients and methods

### Patients

Patients who have suspected T1N0M0 RCC within a cancer network covering 4 hospitals are referred to a specialist tumour board meeting. Each case is discussed by a team comprising urologists, radiologists, pathologists, and oncologists. Both patient and tumour characteristics are considered in formulating treatment recommendations to inform shared decision making with patients. AS, PN, or MWA are all considered as treatment options for cT1a SRMs.

### Procedure

Percutaneous renal mass biopsy (RMB) is routinely performed in advance of MWA treatment to confirm a histological diagnosis of RCC prior to ablation.

Experienced interventional radiologists at the regional tertiary care centre perform CT-guided percutaneous MWA. The patient is first placed under general anaesthesia. This ensures comfort for the patient and enhances the procedural accuracy by enabling temporary pauses in ventilation to reduce movement.

**Figure 1.**
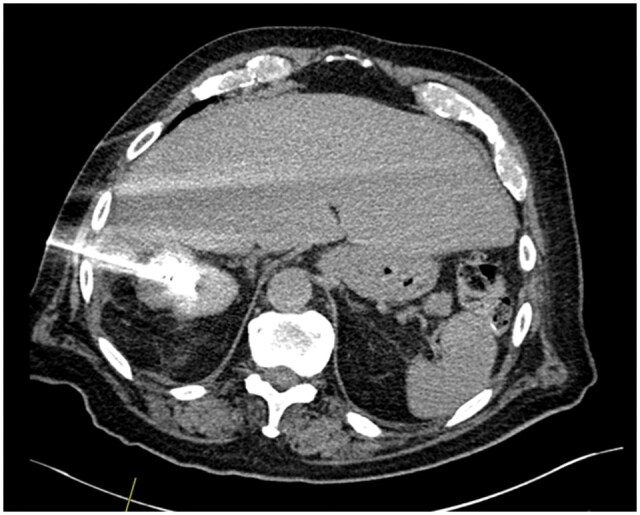
Axial computerized tomography slice taken during the procedure demonstrates the microwave ablation probe with the tip situated within a right upper pole renal mass.

The microwave probe is positioned using CT guidance. Hydro-dissection, which involves the introduction of saline into the abdominal cavity to displace adjacent bowel, can be used in certain cases to ensure a safer needle trajectory. Helical CT scans confirm guide probe positioning towards the centre of the tumour (Figure 1). Ablation is then performed, with power and time calculated in advance. Larger tumours may require multiple overlapping ablations. The needle track is ablated as the probe is withdrawn to minimize haemorrhage or tumour seeding risk. Once the procedure is complete, the patient is moved to recovery and then to a surgical ward where they are observed. Local protocol is for discharge the following day.

During the first 18 months of this service, routine day 1 post-procedure CT imaging was performed. After 18 months, once the procedural learning curve was established, no routine post-procedure CT imaging was performed unless there was a specific clinical concern such as post-ablation bleeding.

### Follow-up

CT surveillance following MWA is protocolized and is organized by the referring hospital at 3, 6, and 12 months and annually thereafter. Any findings suggestive of recurrence or new disease are re-referred for discussion by the specialist tumour board meeting.

### Data collection and analysis

All MWAs performed for suspected RCC were prospectively recorded on a database. Patient, tumour, procedure characteristics, and oncological outcomes and complications were recorded. Local and metastatic recurrences were identified using follow-up imaging and patient notes, in conjunction with radiological interpretation at the specialist tumour board meeting. Patients with benign histology were not included in recurrence calculations.

The follow-up duration was calculated as the time between the MWA and the most recent cross-sectional imaging. Loss to follow-up was defined as any patient who did not have follow-up imaging for more than 12 months following ablation (excluding patients who were discharged from follow-up or patients who had an ablation within 12 months of the data collection period). R.E.N.A.L. nephrometry scores were calculated using the most recent pre-ablation cross-sectional imaging available.^12^ Where available the latest pre-procedure and earliest post-procedure renal function test results were used to calculate median renal function change.

The availability of electronic healthcare records for patients across the region facilitated capture of follow-up data for a large majority of patients. Data were collected up until April 2024.

No formal ethical approval was required for this service evaluation. All patients undergoing MWA treatment had informed consent as per standard of care.

## Results

### Demographics and tumour characteristics

Between October 2016 and April 2024, 209 MWAs were performed, including for 5 patients who had 2 MWAs performed for distinct RCCs during the same anaesthetic episode and 6 patients who underwent repeat ablations following local recurrence. These were recorded and analysed as separate MWA procedures.

The median (range) age was 69 (33-92) years, 73% were male, and the median (interquartile range [IQR]) Charlson Comorbidity Index (CCI) was 1 (0-2). Median (IQR) age-adjusted CCI was 3 (2-5). Tumour characteristics are detailed in Table 1.

**Table 1.** Laterality, size, and position of renal tumours treated with microwave ablation.

Laterality	*n*	%
Left	103	49
Right	106	51
Max tumour diameter		
Median (IQR)	26 (20-32) mm	
<10 mm	3	1
10-20 mm	56	27
20-30 mm	89	43
30-40 mm	47	22
40-50 mm	14	7
Tumour position		
Upper pole	52	25
Interpolar	81	39
Lower pole	75	36
Partial nephrectomy scar	1	0

Abbreviation: IQR = interquartile range.

The biopsy rate was 92%. Histology results can be seen in Table 2. The median R.E.N.A.L. (Radius, Exophytic/Endophytic, Nearness, Anterior/Posterior, Location) nephrometry score was 8 (IQR 6-9, range 4-12).

**Table 2.** Histological morphology of renal tumours treated with microwave ablation.

Morphology and grade	*n*	%
Total with histology	192	92
Total clear cell	110	57
Grade 1 clear cell	16	8
Grade 2 clear cell	68	33
Grade 3 clear cell	14	7
Grade 4 clear cell	2	1
Clear cell, no reported grade	10	5
Total papillary	35	18
Papillary type 1	16	8
Papillary type 2	5	2
Papillary not otherwise specified	14	7
Total other	47	24
Chromophobe	11	5
Oncocytoma	14	7
Oncocytoma/chromophobe mixed type	3	1
Eosinophilic	1	0
Mucinous tubular/spindle cell	2	1
RCC not otherwise specified	1	0
Indeterminate	5	2
Angiomyolipoma	1	0
Other benign tissue	8	4
Histology not reported	1	0

### Procedure and follow-up

Median (IQR) follow-up duration was 37 (15.3-59.4) months. Data were prospectively recorded over a period of 7.5 years. About 94.7% (198) of patients had at least 12 months of follow-up imaging available.

Technical ablation characteristics are listed in Table 3.

**Table 3.** Total ablation time, energy delivered, number of ablations, and recorded use of track ablation during microwave ablation for renal cancer.

Technical ablation characteristics	*n*	%
Total ablation time (min)		
0-5	131	63
6-10	72	34
11-15	0	0
16-20	1	0
>20	0	0
Not reported	5	2
Total energy delivered (kJ)		
0-25	37	18
26-50	66	32
51-75	65	31
76-100	25	12
>100	11	5
Not reported	5	2
Track ablation used		
Yes	102	49
No/not recorded	107	51
Number of ablations during primary procedure		
1	108	52
2	78	37
3	20	10
4	3	1
5	0	0

Use of hydro-dissection was documented in 38 ablations (18.2%). There was 1 recorded use of pyeloperfusion (0.48%).

About 93% (195) of patients had a hospital stay of 1 night. Two patients (1%) were discharged the same day. Length of stay ≥2 nights (12 patients, 6%) was due to social reasons (*n* = 1), to facilitate reloading with warfarin (*n* = 1), post-procedure pain (*n* = 5), lower respiratory tract infection (*n* = 2), and haematoma (*n* = 3). The longest stay was 10 nights (due to pneumonia).

Pre- and post-procedure renal function tests were available for 57% of patients. Median (IQR) eGFR change for those with results available was 0% (−10% to +5%).

### Recurrence

There were 11 local recurrences (5.9%) at a median time of 21.5 months (IQR 10.5-34.9, range 6.6-43.3 months). Five patients (2.7%) developed metastatic disease at a median time of 14.1 months (IQR 12.2-16.4, range 8.5-40.4 months). Further information about recurrence and metastasis management can be found in Table 4.

**Table 4.** Oncological following microwave ablation of renal cancer, including renal cancer morphology, tumour size, and R.E.N.A.L. nephrometry score alongside time to recurrence and subsequent management.

Oncological outcome	RCC type	Size (mm)	R.E.N.A.L. nephrometry score	Time to recurrence (months)	Management of recurrence
Median (IQR)		32.0 (29.5-37.5)	4 (1.5-8)	21.5 (10.5-34.9), [range 6.6-43.3]	
Local recurrence	Clear cell	24	9	6.6	Re-ablation
	Clear cell	37	8	8.7	Re-ablation
	Papillary type 1	30	1	8.9	Re-ablation
	Clear cell	31	9	12.1	Re-ablation
	Clear cell	24	1	14.5	Re-ablation
	Clear cell	42	2	21.5	Re-ablation (did not proceed)
	Clear cell	38	8	25.4	Radical nephrectomy (tumour thrombus)
	Indeterminate	45	2	33.0	Re-ablation
	Clear cell	32	8	36.7	Surveillance
	Clear cell	29	4	36.8	Radical nephrectomy (tumour thrombus)
	Clear cell	36	1	43.3	Re-ablation
Median (IQR)		25.0 (20 -38)	1 (1-2)	14.1 (12.2-16.4), [range 8.5-40.4]	
Metastatic progression	Clear cell	45	2	8.5	Systemic treatment
	Clear cell	20	1	12.2	Palliation
	Clear cell	25	9	14.1	Palliation
	Clear cell	38	1	16.4	Systemic treatment
	Clear cell	20	1	40.4	Systemic treatment

Abbreviations: RCC = renal cell carcinoma, IQR = interquartile range.

### Complications

There were 34 (16.3%) complications recorded within 30 days of MWA. Twenty-six (12.4%) of these acute complications were Clavien-Dindo grade 1 and included acute kidney injury, vomiting, pain, constipation, mild bleeding from the ablation site, and urinary retention. Six (2.87%) Clavien-Dindo Class 2 complications within 30 days of MWA included pulmonary embolism, urinary tract infection, lower respiratory tract infection, acute kidney injury, peritoneal dialysis peritonitis, and an ablation zone collection managed with antibiotics.

**Figure 2.**
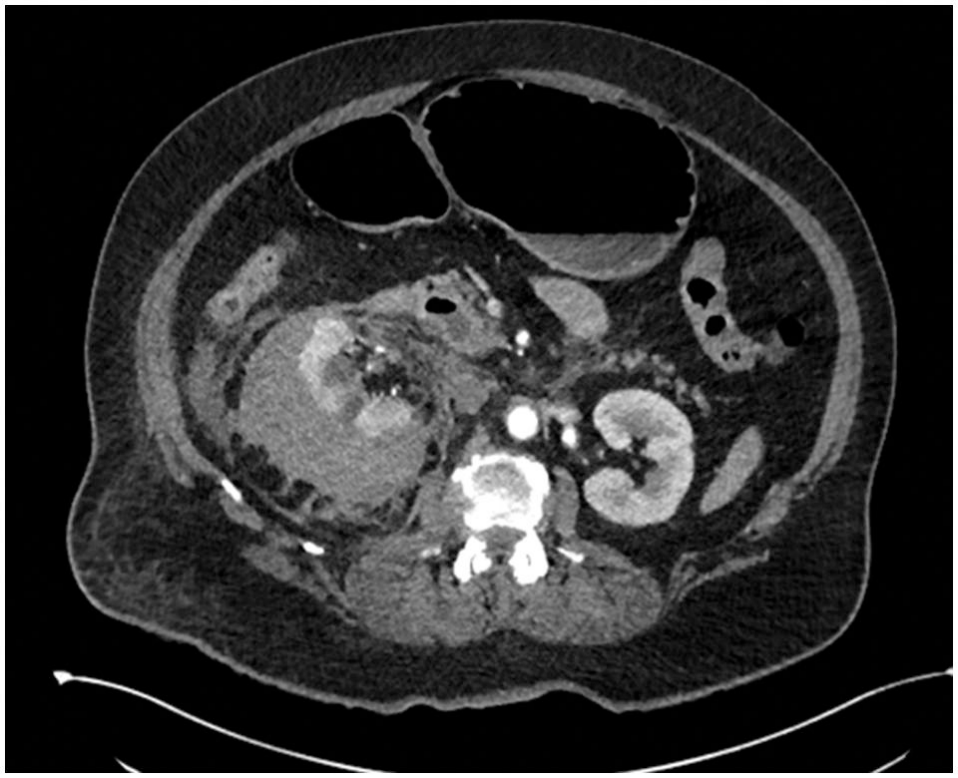
The only grade 4 Clavien-Dindo complication: axial computerized tomography slice with contrast in the arterial phase demonstrating an acute complication of renal microwave ablation. There is a large right sided peri-nephric haematoma causing some compression of the right kidney though this remains well perfused. There is also a small amount of retroperitoneal fluid/blood. No evidence of active haemorrhage was identified on this study, but the patient was taken to intensive care where they received a blood transfusion.

Two (0.96%) acute (within 30 days) and 4 (1.91%) longer-term (beyond 30 days) Clavien-Dindo grade ≥3 complications are listed in Table 5. There was 1 acute Clavien-Dindo grade 4 complication—a large peri-nephric haematoma which required blood transfusion and intensive care unit admission—shown in Figure 2.

**Table 5.** Clavien-Dindo grade ≥3 complications following microwave ablation of renal cancer listed with grade, complication timing, whether re-admission occurred within 30 days, and complication details.

Clavien-Dindo complication grade	Timing of complication (days)	Re-admission within 30 days	Complication details
3a	≤30	Yes	Ablation zone collection requiring ultrasound-guided drainage
4	≤30	No	Large post-procedure haemorrhage, 2 units red blood cell transfusion, intensive care unit admission
3a	>30	No	Ablation zone collection requiring ultrasound-guided drainage
3b	>30	No	Perinephric and abdominal wall abscess requiring surgical incision and drainage
3b	>30	No	Ablation zone collection requiring CT-guided drainage
3b	>30	Yes	Pain, non-functioning kidney on follow-up

Abbreviation: CT = computerized tomography.

Thirteen patients were noted to have incidental ablation site haematomas on post-procedure imaging. In 1 case, the colon was inadvertently punctured during hydro-dissection, and in another the ablation needle sheared off within the kidney and was left *in situ*. Since these patients were asymptomatic and did not require any alteration to routine management, the events were felt not to fall within the Clavien-Dindo grading system and so were not included in the list of complications above.^13^ If these asymptomatic patients are incorporated into the total, the overall acute complication rate is 23.4%.

Eleven patients (5.3%) were re-admitted within 30 days. This was due to neuropathic pain (*n* = 5), constipation (*n* = 1), acute kidney injury (*n* = 1), saddle pulmonary embolism (*n* = 1), peritoneal dialysis peritonitis (*n* = 1), and ablation site collections (*n* = 2).

There were no patient deaths as a complication of MWA.

## Discussion

The current study adds to existing literature one of the largest single cohorts of MWA for SRMs internationally, with a comparatively long follow-up period, very low rate of loss to follow-up, and complete dataset comprising accurate complication and oncological data.

The European Association of Urology (EAU) has recently updated its guidelines^11^ to state that MWA has the best evidence of all the thermal ablative techniques for treatment of SRMs, a significant departure from guidelines as recently as 2022 which described MWA as “experimental”.^14^ This updated position is based on a Chinese study comparing 185 MWAs to 1170 propensity-matched laparoscopic partial nephrectomies, with a median follow-up period of 40.6 months. The authors found no statistically significant differences in oncological outcomes or complication rates between the 2 groups. MWA led to a comparatively smaller reduction in renal function and less procedural blood loss.^15^

Our study has a similarly long follow-up period, but benefits from a larger and more diverse MWA cohort which includes a selection of >40 mm tumours, a greater representation of non-clear-cell renal cancer, and a slightly older cohort with a lower Charlson Comorbidity Index (median 1 vs 4). Our post-operative hospital length of stay was dramatically shorter (median 1 day vs 5.1 days) with comparative or better complication and recurrence rates, supporting our protocol for MWA.

### Population and follow-up

With a median age of 69, the study population demographics are in line with most other literature.^7^^,^^9^ Nearly 95% of patients had at least 12 months of follow-up imaging, and median follow-up period was more than 3 years, in part due to regional sharing of electronic patient records. The completeness of this dataset further strengthens the conclusions around safety and efficacy of MWA for SRMs.

There were more male patients than average RCC prevalence.^16^ The age range of 33-92 and CCI interquartile range of 0-2 indicate that we have performed MWA for a varied assortment of patient characteristics, demonstrating the applicability of MWA to a wide range of cases depending on shared decision making with patients.

### Disease

Biopsy rate is varied in the literature, with some studies describing a rate of 100%^15^^,^^17^ of pre-MWA biopsy whilst others report closer to 85%.^18^ This study cohort, consisting of T1 tumours <5 cm, benefits from a high biopsy rate of 92% alongside a long follow-up period, allowing accurate assessment of oncological outcomes in biopsy-proven RCC. The standard pathway in this centre is for RMB prior to thermal ablation. Where biopsy was not performed this was because biopsy was not procedurally possible, or due to re-treatment of a lesion that has previous histology available. When biopsy was indeterminate (*n* = 5), MWA would proceed based on clinical decision-making weighing the likelihood of an unrepresentative biopsy sample, suggestive radiological appearances, and shared decision making with the patient.

The 23 cases of benign histology were from early in the cohort prior to establishment of a defined biopsy and treatment pathway, when biopsy was frequently performed during the same anaesthetic episode as ablation.

Shared decision making with patients is even more important given the frequency of tumour heterogeneity in SRMs. One study demonstrated only a 68% correlation between pre-operative biopsy and final histology following treatment for oncocytomas, with the discrepancies accounted for by predominantly chromophobe carcinoma and hybrid oncocytoma/chromophobe tumours.^19^

### Intervention

The MWA ablation time of just 4 min reflects the high heating power associated with MWA when compared with other thermal ablative techniques. Track ablation, where the needle path is ablated as the probe is removed, was routinely performed to minimize risk of haemorrhage and tumour seeding.^20^ Adjunctive methods used at the radiologists’ discretion included hydro-dissection and pyeloperfusion. Evidence suggests that in certain cases pyeloperfusion can be protective against iatrogenic heat-induced injury to the collecting system.^21^ Literature comparison between MWA of central compared to peripheral RCC found no difference in outcomes or adverse event profile but noted increased utilization of adjunctive techniques with central tumours to mitigate complications.^14^

The low complication and recurrence rates in the current study suggest that utilization of these adjunctive techniques does not negatively affect safety or oncological outcomes.

### Complications

R.E.N.A.L. nephrometry scores are poorly reported in similar studies, but 1 published ablative series describes 67.6% of their MWA cohort as “low” complexity class, and states a rate of Clavien-Dindo grade 3 or above complications of 5.1%.^17^ A small cohort of 41 MWAs and 82 propensity-matched PNs described approximately 80% of each cohort having a R.E.N.A.L score ≤6, with no serious complications reported in the MWA group.^18^ The median R.E.N.A.L. nephrometry score in the current study was 8 (“moderate”), indicating that tumours of a range of complexity were treated. A score of 8 corresponds with an 11.1% risk of a Clavien-Dindo grade 3 or above complication within 30 days following PN.^22^ Our actual rate of 0.96% is similar or improved compared to other thermal ablative techniques^5^^,^^7^^,^^15^^,^^17^^,^^23–25^ and supports the assertion that MWA is a safe technique.

One small survey of patient reported outcomes after renal ablation including MWA found that nearly 50% of patients thought that they had completely recovered from the ablation on the day after treatment.^26^ In the current study, 94% of patients went home the same or next day following ablation. Very short length of stay post-procedure has obvious advantages both for healthcare providers and for patients.

### Renal function

Renal function testing was available both pre- and post-ablation for 57% of the cohort. Median change in eGFR was 0 mL/min (IQR −10% to +5%). PN series have reported an approximate 30% renal function drop post-surgery,^27^ or development of acute kidney injury or chronic kidney disease.^28^ Although the completeness of the present study’s renal function data is a limitation of this real-world dataset, the trend seen in 57% of patients with a result means that positive inferences can made about those who did not have results available. Given nearly 95% of patients had at least 12 months of follow-up, we can theorize that severe renal function complications (such as dialysis) would also have been appreciated during data collection. The data from the current study therefore supports MWA being safe from the perspective of renal function preservation, which could be particularly important in patients who are vulnerable to further insult.

### Oncological outcomes

The local recurrence rate was 5.9%. One large retrospective cohort study (*n* = 738) of oncological outcomes following PN for predominantly T1 tumours showed a local recurrence rate of 6%,^29^ though rates vary by series with other studies citing T1a tumour recurrence rates of 2.2%.^30^ The local recurrence rate following MWA in the current study is in line with literature rates for PN of pT1a RCC and is similar or better than other reported TA outcomes.

The metastatic progression rate was 2.7%. Background metastatic progression of RCC is strongly dependent on tumour size. Progression rates of approximately 3% for tumours less than 4 cm (T1a) have been evidenced.^31^^,^^32^ Given the comparatively low rate in the current study where median patient follow-up was 37 months, we infer that MWA does not appear to increase the risk of metastasis.

## Conclusion

MWA in our centre is a treatment option with a low complication rate and durable and acceptable long-term oncological outcomes. Given the shorter recovery time and the ability to offer an effective treatment with a lower burden on healthcare resources in terms of procedural time, length of stay in hospital, and need for operating theatre capacity, MWA should be considered as a treatment option for cT1a SRMs.

### Take home message

This cohort series of 209 MWAs with 37-month oncological follow-up and high biopsy rate supports MWA as an effective, safe treatment for SRMs with low rates of disease recurrence, minimal risk of complication, and short recovery period.
